# Iron Overload in Transfusion-Dependent Indonesian Thalassemic Patients

**DOI:** 10.1155/2021/5581831

**Published:** 2021-04-15

**Authors:** Pandji Irani Fianza, Anita Rahmawati, Sri Hudaya Widihastha, Shofura Afifah, Mohammad Ghozali, Andre Indrajaya, Dilli Marayuzan Akbar Pratama, Dimmy Prasetya, Teddy Arnold Sihite, Mas Rizky A. A. Syamsunarno, Djatnika Setiabudi, Suthat Fucharoen, Ramdan Panigoro

**Affiliations:** ^1^Department of Internal Medicine, Division of Hematology and Medical Oncology, Faculty of Medicine, Universitas Padjadjaran/Hasan Sadikin General Hospital, Bandung, West Java, Indonesia; ^2^Research Center of Medical Genetics, Faculty of Medicine, Universitas Padjadjaran, Bandung, West Java, Indonesia; ^3^Doctoral Study Program, Faculty of Medicine, Universitas Padjadjaran, Bandung, West Java, Indonesia; ^4^Department of Biomedical Sciences, Faculty of Medicine, Universitas Padjadjaran, Bandung, West Java, Indonesia; ^5^Department of Cardiology and Vascular Medicine, Faculty of Medicine, Universitas Padjadjaran/Hasan Sadikin General Hospital, Bandung, West Java, Indonesia; ^6^Department of Child Health, Faculty of Medicine, Universitas Padjadjaran/Hasan Sadikin General Hospital, Bandung, West Java, Indonesia; ^7^Thalassemia Research Center, Institute of Molecular Biosciences, Mahidol University, Nakhonpathom, Thailand

## Abstract

Thalassemia is a genetic disease caused by disruption of globin chain synthesis leading to severe anemia and thus regular blood transfusion is necessary. However, there have been known transfusions-related consequences, including iron overload and multi-organ damage. The aims of this study were to evaluate liver and cardiac function in youth and adult transfusion-dependent Indonesian thalassemic patients and to assess its correlation with serum ferritin levels, as well as T2^*∗*^magnetic resonance imaging (MRI). Transfusion-dependent thalassemic (TDT) outpatients (*n* = 66; mean age, 21.5 ± 7.2 years) were carried out for the complete assessment consisting of blood test including liver enzyme and serum ferritin, followed by electrocardiography (ECG) and echocardiography. Subjects were also divided by serum ferritin levels into three groups: < 2500 ng/mL, 2500–5000 ng/mL, and >5000 ng/mL. Additionally, subgroup analysis in patients with T2^∗^ MRI assessment was conducted. In terms of age of first blood transfusion, subjects with ferritin >5000 ng/mL were the youngest among others. The alanine aminotransferase (ALT) levels in group with serum ferritin >5000 ng/mL were significantly higher than those of the group with serum ferritin <2500 ng/mL. Additionally, youth and adult TDT patients whose serum ferritin >5000 ng/mL had significantly lower tricuspid annular plane systolic excursion (TAPSE) when compared with those who had serum ferritin <2500 ng/mL. Similarly, TAPSE in patients with moderate cardiac siderosis based on cardiac T2^∗^ MRI was significantly lower than those without cardiac siderosis. There was significant, but only moderate correlation between serum ferritin and cardiac T2^∗^ MRI. Based on these findings, it is important to routinely monitor iron accumulation-related complications, including liver and cardiac damage in youth and adult TDT patients.

## 1. Introduction

Thalassemia is a hereditary disorder caused by disruption of globin chain synthesis leading to reduced level of hemoglobin [1, 2]. Regular blood transfusion is the main treatment of thalassemia, which restores the important function of hemoglobin as oxygen carrier throughout the body tissues. However, multiple blood transfusions can result in iron overload, which furthermore interfere with the metabolism and lead to tissue and organ damage. Many studies have shown that use of iron-chelating agent resulted in an improved survival in transfusion-dependent thalassemic (TDT) patients. Therefore, regular blood transfusion and iron chelation therapy should be combined adequately [3, 4].

Excess of iron is deposited in vital organs such as heart, liver, spleen, and endocrine organs [5]. Iron overload causes most of the mortality and morbidity associated with thalassemia [6, 7]. Several studies have reported complications found in patients with chronic transfusion, including endocrine dysfunction, hypothyroidism, hyperparathyroidism, hypogonadism, cardiomyopathy, arrhythmia, progressive liver failure, and abnormal kidney function [8, 9]. Centers for Disease Control and Prevention (CDC) reported that complications occurred in adult patients with median age of 31.3 years, with an average age of 4.5 ± 8.2 years at the start of transfusion, and with a median duration of transfusion 18.5 ± 12.3 years [10].

The liver is one of the very susceptible organs commonly affected by iron toxicity. Additionally, cardiac toxicity is the most severe and life-threatening complication of iron overload. Various methods of complications assessment, including serum ferritin level, echocardiography, non-transferrin-bound iron, cardiac T2^∗^ magnetic resonance imaging (MRI), heart rate variability, liver biopsy, and myocardial biopsy, have been used for early detection of iron overload in thalassemia patients. However, controversial evidence and limitations of their use in clinical practice have been reported. Although T2^*∗*^MRI is the recommended non-invasive tool for quantifying iron accumulation, the low cost and widespread availability of serum ferritin make its use indispensable [5]. Studies evaluated iron overload in Indonesian thalassemic patients were mostly performed in children [11] or without organ function assessment [12]. There are still limited studies on the relationship of iron overload and liver, as well as cardiac damage, especially in Indonesian youth and adult TDT patients. Therefore, the objectives of this study were to evaluate liver and cardiac function in Indonesian youth and adult TDT patients and to assess its correlation with serum ferritin levels and T2^*∗*^MRI.

## 2. Methods

### 2.1. Subjects

Youth and adult TDT patients who regularly visited the outpatient clinics at Department of Internal Medicine, Division of Hematology and Medical Oncology, Hasan Sadikin General Hospital, Bandung, for the past 5 years were invited to join this study. Non-probability convenience sampling was used to recruit the patients. The inclusion criteria were as follows: patients who (1) were older than 14 years old, (2) have received transfusion for at least 2 years, (3) were judged capable of completing the survey physically, and cognitively and (4) were capable of understanding spoken and written Indonesian language. Patients who were on acute and/or severe infection determined by history taking and physical examination were excluded because it will interfere with the results of serum ferritin levels [13]. From June to October 2017, upon obtaining written informed consent, each patient with TDT completed questionnaire and blood examination, as well as cardiac function assessment. This study protocol was approved by the Ethics Committee of Faculty of Medicine, Universitas Padjadjaran, Bandung (institutional review board approval number 662/UN6.C10/PN/2017) and conducted in accordance with the Declaration of Helsinki.

Diagnosis of thalassemia was based on the clinical history, molecular diagnosis, and laboratory confirmation. Patients' data were retrieved from medical record and questionnaire. Demographic variables consisting of age, sex, occupation, and marital status were recorded. Questionnaire was used to obtain more personal information of the patients' disease, including age at the start of first transfusion and the interval between blood transfusions. Furthermore, the patients' blood samples were drawn and cardiac examination, including electrocardiography (ECG) and echocardiography, was performed. T2^*∗*^MRI was conducted in Cipto Mangunkusumo National Public Hospital, Jakarta. Therefore, subgroup of patients was randomly selected especially for those who were willing to be checked for MRI in Jakarta.

### 2.2. Blood Examination

Hematological parameters were determined by 6 mL of blood, which was collected through venipuncture from each individual using a disposable syringe under sterile conditions, performed by trained laboratory technician. Automated hematology analyzer (Sysmex XN-®Series™ Hematology Analyzers) was used to analyze parameters, including pre-transfusion hemoglobin. Serum ferritin level was also measured by electrochemiluminescence immunoassay (ECLIA) method using ADVIA Centaur XPT Immunoassay System Siemens Healthineers. Although serum ferritin has limitation to predict cardiac iron overload, it can reliably predict siderosis when it is >2500 ng/mL [13]. Sayed et al. [14] previously defined the iron overload severity by classified the serum ferritin levels into three groups: <2500 ng/mL, 2500–5000 ng/mL, and >5000 ng/mL. Similarly, Eghbali et al. [15] also divided the serum ferritin levels into the same 3 groups.

The levels of liver enzymes, including aspartate aminotransferase (AST) and alanine aminotransferase (ALT), were used to indicate liver function, as injury to acute or chronic liver eventually results in increase in both serum concentrations. To investigate the enzymes level, the tube containing blood sample was put on room temperature and then centrifuged for 15 minutes at 3,000 rpm for further analysis. The assessment of the enzymes was carried out by using Automated Hematology Analyzer Dimension® EXL™ 200.

### 2.3. Cardiovascular Function Test

ECG and echocardiography to examine cardiovascular function were performed seven days after blood transfusion. Therefore, cardiac alterations found in the assessment could be assumed caused by iron overload and not by anemia. Standard 9-lead ECG was used and the cardiologist analyzed the result. In addition, complete resting two-dimensional and tissue Doppler echocardiography were performed to the patients in the left lateral decubitus position by the same experienced echo cardiographer, by using general electric vivid S6 cardiovascular ultrasound system equipped with 1.5–3.6 MHz Cardiac Section Probe M4S-RS. The diagnostic criteria were according to American Society of Echocardiography [16]. The examinations included left ventricular end-diastolic diameter (LVEDD), left ventricular end-systolic diameter (LVESD), and posterior wall (PW) thickness, all expressed in millimeters. From apical four-chamber view, left ventricular end-systolic volume (LVESV), left ventricular end-diastolic volumes (LVEDV), and left ventricular stroke volume (LVSV) were assessed. Left ventricular ejection fraction (LVEF) and fractional shortening (FS) were measured to see left ventricular function. Left ventricular filling was evaluated by the tissue Doppler echocardiography, from the apical four-chamber view, velocities in early (*E*) and late (*A*) diastole were recorded, and also *E*/*A* ratio and deceleration time (DT) of E-wave were calculated. Mean values of early (*E′*) myocardial velocities were calculated to find the *E*/*E*′ ratio. Furthermore, the left atrial maximum volume index (LAVI) and mean pulmonary artery (PA) pressure were calculated.

### 2.4. T2^∗^ MRI Assessment

The T2^∗^ MRI assessments of subgroup of patients were conducted at the Department of Radiology, Cipto Mangunkusumo National Public Hospital, Jakarta. All cardiac and liver T2^∗^ images were obtained at 1.5 Tesla MR scanner (Siemens Avanto Germany). Cardiac T2^∗^ time was obtained from the region of interest at ventricular septum to avoid artifact and liver T2^∗^ time was obtained from 10 mm slice through the center of the liver and analyzed via computer software [11]. Cardiac T2^∗^ value was considered as mild (15–20 ms), moderate (10–15 ms), and severe (<10 ms). Liver T2^∗^ value was considered as mild (3.8–11.4 ms), moderate (1.8–3.8 ms), and severe (<1.8 ms) [17].

### 2.5. Statistical Analysis

Data were presented as means ± standard deviation or median (interquartile range) for continuous variables and percentages for categorical variables. Variables were assessed for normality by Kolmogorov-Smirnov or Shapiro-Wilk test. Discrete variables were compared with the chi-square test (Kolmogorov-Smirnov test when appropriate) and continuous variables with one-way analysis of variance (ANOVA) and multiple comparisons with Tukey post hoc test (Kruskal-Wallis test when appropriate). Differences between two groups were analyzed using Student's *t*-test or Mann–Whitney *U* test when appropriate. Correlation coefficients for liver and cardiac function and serum ferritin, as well as T2^∗^ MRI, were obtained using Pearson product-moment correlations or Spearman's rho correlation coefficient test if appropriate. A *p* value < 0.05 was considered significant and all tests were two-tailed. Data were analyzed using SPSS 24.0 (SPSS, Chicago, IL, USA).

## 3. Results

### 3.1. Patient Characteristics

Patient characteristics for the total group are given in Table 1. The total group consisted of 25 men and 41 women (mean age, 21.5 ± 7.2 years). The age range in this study was 15–53 years. The mean age of the first blood transfusion was 60.1 ± 86.8 months with the mean transfusion interval 5.6 ± 6.3 weeks. Deferasirox was the most commonly used iron-chelating agent (46.4%), followed by deferiprone (41.1%). The mean hemoglobin before blood transfusion was 7.2 ± 1.7 g/dL.

The mean serum ferritin was 4414.5 ± 3165.2 ng/mL. Based on previous study [14, 15], the characteristics among three groups of serum ferritin levels (<2500 ng/mL, 2500–5000 ng/mL, and >5000 ng/mL) were compared, as shown in Table 2. Significant differences were observed in age of the first blood transfusion (*p* ≤ 0.01), but not in other characteristics. With respect to the patients with different iron-chelating agents, there were no significant differences in number of patients among the three groups.

### 3.2. Liver Function Test

The results of liver function test stratified by serum ferritin levels are demonstrated in Table 2. The mean of AST and ALT in those with serum ferritin >5000 ng/mL tended to be the highest among the three groups (*p* ≤ 0.1 and *p* ≤ 0.1, respectively). In two groups comparison, ALT levels in group with serum ferritin >5000 ng/mL were significantly higher than those of the group with serum ferritin <2500 ng/mL (60.9 ± 43.6 ng/mL vs 37.5 ± 19.3 ng/mL, *p* < 0.05). By using T2^∗^ MRI, subgroup of patients was analyzed to examine the relationship of liver function in the subgroup. Although not reaching statistical significance, liver enzymes including AST and ALT seem to be higher in those with liver siderosis, as shown in Table 3.

**Figure 1:**
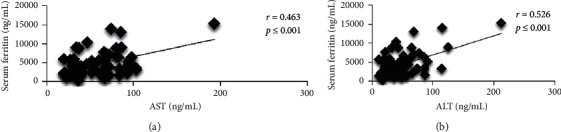
Correlation between serum ferritin and liver enzyme. (a) Correlation between serum ferritin and aspartate aminotransferase (AST). (b) Correlation between serum ferritin and alanine aminotransferase (ALT).

**Figure 2:**
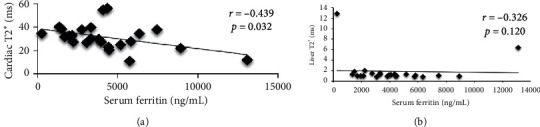
Correlation between serum ferritin and T2^∗^ MRI. (a) Correlation between serum ferritin and cardiac T2^∗^. (b) Correlation between serum ferritin and liver T2^∗^.

In terms of correlation analysis, as shown in Figure 1, AST was positively correlated with serum ferritin (*r* = 0.463, *p* < 0.05). In addition, there was significantly strong and positive correlation between ALT and serum ferritin (*r* = 0.526, *p* ≤ 0.01) in youth and adult TDT patients.  Figure 2 shows that there was no correlation between serum ferritin and liver T2^∗^ MRI in subgroup analysis.

### 3.3. Cardiac Function Test

Table 2 lists comparison results of echocardiography parameters and ECG among the three groups. Regarding echocardiography parameters, only left ventricular posterior wall diastolic diameter (LVPWDD, 7.7 ± 1.1 mm) and tricuspid annular plane systolic excursion (TAPSE, 21.1 ± 2.9 mm) were significantly lower among three groups (*p* < 0.05). In two comparisons, youth and adult TDT patients whose serum ferritin >5000 ng/mL had significantly lower LVPWDD (7.7 ± 1.1 mm vs 9.0 ± 1.9 mm, *p* < 0.05), left ventricular stroke volume (LVSV, 57.3 ± 10.2 mL vs 66.4 ± 14.2 mL, *p* < 0.05), and TAPSE (21.1 ± 2.9 mm vs 24.0 ± 3.8 mm, *p* < 0.05) compared with those with serum ferritin <2500 ng/mL. In line with this, subgroup analysis presented in Table 4 shows the relationships between presence of cardiac siderosis based on T2^∗^ MRI and cardiac function. TAPSE in patients with moderate cardiac siderosis was significantly lower than those without cardiac siderosis (17.0 ± 2.8 mm vs 23.8 ± 3.6 mm, *p* < 0.05). There were some tendencies regarding the relationships of LVPWDD, LVSV, and LVFS among patients with and without moderate cardiac siderosis (*p* ≤ 0.1).

With regard to ECG, there were no significant differences in ECG results among the three groups. Most of youth and adult TDT patients have normal ECG findings. T-wave inversion (31.7%) was the most common ECG abnormality, followed by tachycardia (9.5%) and left ventricular hypertrophy (6.3%). After being stratified by presence of cardiac siderosis based on T2^∗^ MRI findings, there was significant difference on ECG changes in TDT patients as shown in Table 4. On the other hand, there was significant, but moderate correlation between serum ferritin and cardiac T2^∗^ MRI as shown in Figure 2.

## 4. Discussion

Serum ferritin remains an inexpensive and easily available tool for assessment of iron overload regardless of its limitation, but T2^∗^ MRI still becomes a recommended strategy to evaluate iron overload [13, 18]. Prior works have demonstrated some correlation between liver or cardiac function tests and serum ferritin or T2^∗^ MRI [11, 19–23]. However, most of those studies included pediatric to young adult thalassemic patients. In this study, we tested the extent to which serum ferritin levels as well as T2^∗^ MRI correlated with liver and cardiac function among youth and adult TDT patients.

In this study, age of the first blood transfusion was found significantly associated with serum ferritin levels. The earlier the age at the start of blood transfusion, the higher the serum ferritin levels. Mishra and Tiwari [24] reported that serum ferritin level can also be increased due to frequency of blood transfusion and the age of the patient. Serum ferritin is commonly used to monitor patients with transfusion iron overload as it often is used to adjust or switch chelation regimens. Appropriate chelation therapy has been shown to reduce liver and myocardial iron overload [18].

In our study, most of patients were given deferasirox followed by deferiprone as their iron chelation therapy. It is known that deferasirox and deferiprone offer an important treatment option for people with thalassemia and secondary iron overload [25–28]. Deferoxamine was administered only in small number of patients due to its negative impact on patient's quality of life, such as discomfort and time-consuming in its administration. Combination of two chelation agents was the smallest number because of patient's limitation and problem with insurance coverage. Similar to our study, deferasirox is more often used as an oral iron-chelator followed by deferiprone in Europe. Although information on compliance was not systematically collected in our study, the lack of compliance to iron chelation therapy possibly explains high levels of serum ferritin in several patients [29].

Our study demonstrated higher AST and ALT levels occurred with increased serum ferritin concentration. In line with this, Al-Moshary et al. [19] have discovered similar correlation in children to young adult thalassemic patients. In addition, a study conducted in Bangladesh has reported that the higher levels of serum AST and ALT in beta-thalassemia patients indicate an abnormal muscle and liver function [30]. These finding were also found in Jordan; a positive correlation between serum ALT and AST concentrations and serum ferritin levels in beta-thalassemia patients compared to controls was found [31]. Previous studies conducted in children and adolescences showed correlation between liver T2^*∗*^MRI and serum ferritin, as well as liver enzymes [11, 17]. However, in this study, only similar trends were found although not reaching statistical significance, probably because of the small sample sizes, which affect the power of analysis.

Cardiac dysfunction secondary to iron overload in thalassemia patients may start early in life although clinical signs are not observed in most patients [32]. Regarding cardiac iron concentration, the T2^∗^ MRI inspection remains the best way to detect cardiac hemosiderosis [33]. In this study, the correlation between cardiac T2^∗^ MRI and serum ferritin was not strong. Similarly, other studies also found weak or no correlation between cardiac T2^∗^ and serum ferritin [11, 17]. It indicated the importance of cardiac T2^∗^ MRI to be routinely screened to detect cardiac overload.

ECG abnormalities reported in thalassemia patients have been documented in previous studies [34–37]. The most common abnormalities presented in this study were in accordance to Ramazzotti et al. [37], which reported T-wave inversion as the most common ECG changes. These findings suggested that repolarization were affected by myocardial iron deposit. The changes in repolarization are consistent with impairment of delayed rectifier potassium channels observed in animal models of iron overload [35]. In addition, presence of T-wave inversion had been shown to be related to higher risk for severe cardiac events [38].

In this study, LVPWDD had relationship with serum ferritin levels. It may imply that diastolic dysfunction may occur with the increased of serum ferritin, reflecting an alteration in diastolic property most probably caused by iron accumulation in the heart. Based on natural history, diastolic dysfunction generally appears before systolic dysfunction [36, 39]. Similarly, Sayed et al. [14] reported that diastolic functions were significantly impaired in patients with serum ferritin >5000 ng/mL. Diastolic dysfunction secondary to iron overload can be explained by the initial phase of the structural heart alterations that iron can affect all cardiac structures including papillary muscles, conduction system, and pericardium [40]. The cardiac abnormalities may also be related to poor compliance of iron-chelator [41].

In terms of ventricular systolic function evaluation, decreased LVSV in patients with higher serum ferritin levels suggested some degree of left ventricular dysfunction. In study by Rodrigues et al. [40], left ventricular dysfunction was indicated by the lower value of the percentage of systolic posterior wall thickening. Another interesting finding in our study is that TAPSE was significantly correlated with serum ferritin levels and cardiac T2^∗^ MRI. It may indicate that iron deposition in myocardium in patients with cardiac siderosis could affect right heart function [21].

There were some limitations in this study. This was a cross-sectional study, which does not allow for the inference of cause and effect relationship. Furthermore, a greater number of subjects are necessary in any further study. Although T2^∗^ MRI is the best way to quantify iron accumulation, the low cost and widespread availability of serum ferritin make its use necessary [5].

In conclusion, our results indicated that serum ferritin level and T2^∗^ MRI value in youth and adult TDT patients have relationships with liver and cardiac function. It is necessary to re-evaluate the chelation therapy in patients with higher serum ferritin levels, including the compliance to chelation therapy. In addition, access of T2^∗^ MRI should be provided in area with high prevalence of TDT. Based on these findings, it is important to routinely monitor any possible complications, including liver and cardiac damage in youth and adult patients with TDT. Early detection and therefore timely treatment of such complications could improve the quality of life of these patients.

## Figures and Tables

**Table 1:** Patient baseline characteristics for the total sample and stratified by serum ferritin level classification.

	Serum ferritin (ng/mL)				
	<2500 (*n* = 22)	2500–5000 (*n* = 20)	>5000 (*n* = 24)	Total (*n* = 66)	*p* value
*Demographic*					
Age, years (mean ± SD)	22.6 ± 8.9	22.6 ± 8.0	19.6 ± 3.7	21.5 ± 7.2	0.275
Sex, *n* (%)					0.592
Male	10 (45.5)	6 (30.0)	9 (37.5)	25 (37.9)	
Female	12 (54.5)	14 (70.0)	15 (62.5)	41 (62.1)	

*Clinical factors*					
Age of first transfusion, months (mean ± SD)	109.2 ± 119.8	50.4 ± 61.5	23.2 ± 35.7^*∗*^	60.1 ± 86.8	0.003
Transfusion interval, weeks (mean ± SD)	6.7 ± 10.3	6.0 ± 3.1	4.3 ± 1.9	5.6 ± 6.3	0.421
Type of chelation therapy, *n* (%)					
Deferoxamine monotherapy	1 (5.3)	2 (11.1)	1 (5.3)	4 (7.1)	0.261
Deferiprone monotherapy	11 (57.9)	7 (38.9)	5 (26.3)	23 (41.1)	
Deferasirox monotherapy	7 (36.8)	7 (38.9)	12 (63.2)	26 (46.4)	
Deferoxamine and deferasirox	0 (0.0)	2 (11.1)	1 (5.3)	3 (5.4)	
Pre-transfusion Hb, g/dL (mean ± SD)	6.9 ± 1.2	7.3 ± 1.4	7.4 ± 2.3	7.2 ± 1.7	

Hb: hemoglobin, ^*∗*^*p* < 0.01 compared with serum ferritin <2500.

**Table 2:** Liver enzyme and cardiac function parameters stratified by serum ferritin level classification.

	Serum ferritin (ng/mL)			
	<2500 (*n* = 22)	2500–5000 (*n* = 20)	>5000 (*n* = 24)	*p* value
*Liver enzymes, ng/mL (mean* ± *SD)*				
AST	44.8 ± 21.9	54.6 ± 2.0	65.3 ± 35.9	0.066
ALT	37.5 ± 19.3	50.5 ± 25.5	60.9 ± 43.6^*∗*^	0.051
*Cardiac function parameters (mean* ± *SD)*				
Echocardiography				
LVPWDD (mm)	9.0 ± 1.9	8.8 ± 1.5	7.7 ± 1.1^*∗*^	0.017
LVPWSD (mm)	13.6 ± 1.8	14.8 ± 4.5	13.5 ± 2.7	0.312
LVEDV (mL)	145.5 ± 14.3	96.0 ± 21.5	88.8 ± 19.0	0.066
LVESV (mL)	36.8 ± 11.5	32.8 ± 9.7	31.5 ± 10.7	0.221
LVSV (mL)	66.4 ± 14.2	63.1 ± 13.3	57.3 ± 10.2^*∗*^	0.054
LVEF (%)	64.7 ± 4.8	66.3 ± 4.5	65.2 ± 5.2	0.552
LVFS (%)	35.4 ± 3.9	36.6 ± 3.3	35.6 ± 3.9	0.580
LV diastolic function E/A ratio (m/s)	1.6 ± 0.4	1.6 ± 0.3	1.6 ± 0.3	0.886
LV diastolic function DT (ms)	168.0 ± 43.3	155.5 ± 27.2	154.0 ± 25.6	0.304
LV diastolic function E/E′	9.7 ± 2.7	13.5 ± 18.5	12.4 ± 13.1	0.636
LV diastolic function IVRT (ms)	70.5 ± 8.9	71.3 ± 5.1	64.5 ± 17.2	0.262
LV diastolic function LAVI (mL/m^2^)	33.9 ± 17.3	28.2 ± 5.4	27.3 ± 6.8	0.130
Mean PA pressure (mmHg)	18.6 ± 10.1	18.6 ± 8.3	20.2 ± 8.5	0.799
TAPSE (mm)	24.0 ± 3.8	23.4 ± 2.8	21.1 ± 2.9^*∗*^	0.011
Electrocardiography, *n* (%)				0.958
Normal	11 (52.4)	8 (44.4)	10 (41.7)	
Tachycardia	2 (9.5)	0 (0.0)	4 (16.7)	
T-wave inversion	3 (14.3)	10 (55.6)	7 (29.2)	
Left ventricular hypertrophy	2 (9.5)	0 (0.0)	2 (8.3)	
RBBB	2 (9.5)	0 (0.0)	0 (0.0)	
Arrhythmia	1 (4.8)	0 (0.0)	0 (0.0)	
Supraventricular extra systole	0 (0.0)	0 (0.0)	1 (4.2)	

^*∗*^ *p* < 0.05 compared with serum ferritin <2500, AST: aspartate aminotransferase; ALT: alanine aminotransferase; LVPWDD: left ventricular posterior wall diastolic diameter; LVPWSD: left ventricular posterior wall systolic diameter; LVEDV: left ventricular end-diastolic volume; LVESV: left ventricular end-systolic volume; LVSV: left ventricular stroke volume; LVEF: left ventricular ejection fraction; LVFS: left ventricular fractional shortening; LV: left ventricle; DT: deceleration time; IVRT: isovolumic (or isovolumetric) relaxation time; LAVI: left atrial volume index; PA: pulmonary artery; TAPSE: tricuspid annular plane systolic excursion; RBBB: right bundle branch block.

**Table 3:** Liver function stratified by presence of liver siderosis based on T2^*∗*^ MRI.

	T2^*∗*^ ≥ 1.8 (*n* = 4)	T2^*∗*^ < 1.8 (*n* = 20)	*p* value
Age (years), median (IQR)	23.5 (18.5, 28.5)	20.0 (18.0, 21.75)	0.185
Serum ferritin (ng/mL), median (IQR)	1850.45 (563.1, 10363.4)	4008.0 (2815.8, 5598.5)	0.245
Liver enzymes (ng/mL), median (IQR)			
AST	60.0 (40.0, 80.8)	39.0 (33.3, 80.0)	0.509
ALT	55.0 (26.0, 65.3)	41.5 (29.8, 65.5)	0.877

IQR: interquartile range, AST: aspartate aminotransferase; ALT: alanine aminotransferase.

**Table 4:** Cardiac function stratified by presence of cardiac siderosis based on T2^*∗*^ MRI.

	None (T2^*∗*^ > 20 ms, *n* = 22)	Moderate (T2^*∗*^ 10–15 ms, *n* = 2)	*p* value
Age, years (mean ± SD)	20.9 ± 4.0	22.5 ± 2.1	0.247
Serum ferritin, ng/mL (mean ± SD)	3732.9 ± 2096.8	9413.0 ± 5188.7	0.060
Cardiac T2^*∗*^ (ms)	33.3 ± 9.2	11.2 ± 0.7	0.022
Echocardiography			
LVPWDD (mm)	8.7 ± 1.5	7.2 ± 0.0	0.059
LVSV (mL)	64.7 ± 11.3	48.5 ± 6.4	0.067
LVEF (%)	66.2 ± 4.7	60.0 ± 2.8	0.058
LVFS (%)	36.5 ± 3.5	31.5 ± 2.1	0.050
LV diastolic function E/A ratio (m/s)	1.7 ± 0.3	1.5 ± 0.4	0.531
LV diastolic function DT (ms)	159.7 ± 43.3	165.0 ± 14.1	0.497
LV diastolic function E/E′	15.9 ± 21.3	12.2 ± 4.1	0.347
LV diastolic function IVRT (ms)	64.7 ± 17.6	74.0 ± 0.0	0.348
LV diastolic function LAVI (mL/m^2^)	29.3 ± 6.6	28.1 ± 3.0	0.870
Mean PA pressure, mmHg	17.7 ± 8.5	27.0 ± 11.3	0.154
TAPSE, mm	23.8 ± 3.6	17.0 ± 2.8	0.040
Electrocardiography, *n* (%)			0.037
Normal	12 (60.0)	0 (0.0)	
Tachycardia	0 (0.0)	1 (50.0)	
T-wave inversion	6 (30.0)	0 (0.0)	
Left ventricular hypertrophy	0 (0.0)	1 (50.0)	
RBBB	1 (5.0)	0 (0.0)	
Arrhythmia	1 (5.0)	0 (0.0)	

LVPWDD: left ventricular posterior wall diastolic diameter; LVPWSD: left ventricular posterior wall systolic diameter; LVEDV: left ventricular end-diastolic volume; LVESV: left ventricular end-systolic volume; LVSV: left ventricular stroke volume; LVEF: left ventricular ejection fraction; LVFS: left ventricular fractional shortening; LV: left ventricle; DT: deceleration time; IVRT: isovolumic (or isovolumetric) relaxation time; LAVI: left atrial volume index; PA: pulmonary artery; TAPSE: tricuspid annular plane systolic excursion; RBBB: right bundle branch block.

## Data Availability

The data used to support the findings of this study are available from the corresponding author upon request.
